# Relative Impact of Complement Receptors CD21/35 (Cr2/1) on Scrapie Pathogenesis in Mice

**DOI:** 10.1128/mSphereDirect.00493-17

**Published:** 2017-11-22

**Authors:** Sarah J. Kane, Eric Swanson, Elizabeth O. Gordon, Savannah Rocha, Heather R. Bender, Luke R. Donius, Adriano Aguzzi, Jonathan P. Hannan, Mark D. Zabel

**Affiliations:** a Prion Research Center, Department of Microbiology, Immunology, and Pathology, College of Veterinary Medicine and Biomedical Sciences, Colorado State University, Fort Collins, Colorado, USA; b Dana-Farber Cancer Institute, Medical Oncology, Boston, Massachusetts, USA; c Institute of Neuropathology, University of Zürich, Zürich, Switzerland; d Department of Medicine, University of Colorado School of Medicine, Aurora, Colorado, USA; University of Michigan—Ann Arbor; University of Edinburgh; Hokkaido University

**Keywords:** complement receptors, prions, spleen

## Abstract

Complement receptors 1 and 2 (CR1/2 or CD35/CD21) recognize complement-opsonized antigens to initiate innate and adaptive immunity, respectively. CD35 stimulates phagocytosis on macrophages and antigen presentation on follicular dendritic cells (FDCs). CD21 helps activate B cells as part of the B cell coreceptor with CD19 and CD81. Differential splicing of transcripts from the mouse *Cr2* gene generates isoforms with both shared and unique complement binding capacities and cell-type expression. In mouse models, genetic depletion of *Cr2* causes either a delay or complete prevention of prion disease, but the relative importance of CD35 versus CD21 in promoting prion disease remains unknown. Here we show that both isoforms act as high-affinity cell surface prion receptors. However, mice lacking CD21 succumbed to terminal prion disease significantly later than mice lacking CD35 or wild-type and hemizygous mice. CD21-deficient mice contained fewer splenic prions than CD35 knockout mice early after infection that contributed to delayed prion neuroinvasion and terminal disease, despite forming follicular networks closer to proximal nerves. While we observed no difference in B cell networks, PrP^C^ expression, or number of follicles, CD21-deficient mice formed more fragmented, less organized follicular networks with fewer Mfge8-positive FDCs and/or tingible body macrophages (TBMφs) than wild-type or CD35-deficient mice. *In toto*, these data demonstrate a more prominent role for CD21 for proper follicular development and organization leading to more efficient lymphoid prion replication and expedited prion disease than in mice expressing the CD35 isoform.

**IMPORTANCE** Mammalian prion diseases are caused by prions, unique infectious agents composed primarily, if not solely, of a pathologic, misfolded form of a normal host protein, the cellular prion protein (PrP^C^). Prions replicate without a genetic blueprint, but rather contact PrP^C^ and coerce it to misfold into more prions, which cause neurodegeneration akin to other protein-misfolding diseases like Alzheimer’s disease. A single gene produces two alternatively spliced mRNA transcripts that encode mouse complement receptors CD21/35, which promote efficient prion replication in the lymphoid system and eventual movement to the brain. Here we show that CD21/35 are high-affinity prion receptors, but mice expressing only CD21 die from prion disease sooner than CD35-expressing mice, which contain less prions early after infection and exhibit delayed terminal disease, likely due to their less organized splenic follicles. Thus, CD21 appears to be more important for defining splenic architecture that influences prion pathogenesis.

## Abstract (precis)

Mammalian prion diseases are caused by prions, unique infectious agents composed primarily, if not solely, of a pathologic, misfolded form of a normal host protein, the cellular prion protein (PrP^C^). Prions replicate without a genetic blueprint, but rather contact PrP^C^ and coerce it to misfold into more prions, which cause neurodegeneration akin to other protein-misfolding diseases like Alzheimer’s disease. A single gene produces two alternatively spliced mRNA transcripts that encode mouse complement receptors CD21/35, which promote efficient prion replication in the lymphoid system and eventual movement to the brain. Here we show that CD21/35 are high-affinity prion receptors, but mice expressing only CD21 die from prion disease sooner than CD35-expressing mice, which contain less prions early after infection and exhibit delayed terminal disease, likely due to their less organized splenic follicles. Thus, CD21 appears to be more important for defining splenic architecture that influences prion pathogenesis.

## INTRODUCTION

Mammalian prion diseases are caused by prions, unique infectious agents thought to be composed primarily, if not solely, of PrP^Sc^, a pathologic, misfolded form of a normal host protein, the cellular prion protein (PrP^C^). The prion and/or PrP^Sc^, first identified in the sheep prion disease scrapie (1), replicates without a genetic blueprint, but rather contacts PrP^C^ and coerces it to misfold into more PrP^Sc^. Prions cause neurodegeneration akin to other protein-misfolding diseases like Alzheimer’s disease.

The complement system consists of over 30 soluble and cell-surface proteins which serve three main functions: (i) to opsonize extracellular pathogens to trigger phagocytosis, (ii) to create the membrane attack complex (MAC) on pathogen cell surfaces to provoke cell lysis, and (iii) to reduce the threshold for B cell activation upon simultaneous engagement of B cell receptor and complement receptor 2. While complement activation involves a largely nonspecific recognition of proteins and lipids, it links the fast-acting innate immune system with the highly specific, long-term, memory-inducing, adaptive immune system. During peripheral prion infection, complement facilitates the early spread and propagation of PrP^Sc^ to and within the lymphoreticular system (LRS). Mice lacking complement proteins C3, C4, C1q, and CD21/35 exhibit delayed PrP^Sc^ accumulation and prion disease onset (2–4). Interestingly, in a mouse model of chronic wasting disease, genetic depletion of complement receptors CD21/35 (5) completely protected mice from infection, whereas depletion of the receptors’ ligand, C3, delayed but did not entirely prevent disease (6). These data strongly suggest CD21 or -35 impacts disease independently of its natural ligand, C3.

**FIG 1:**
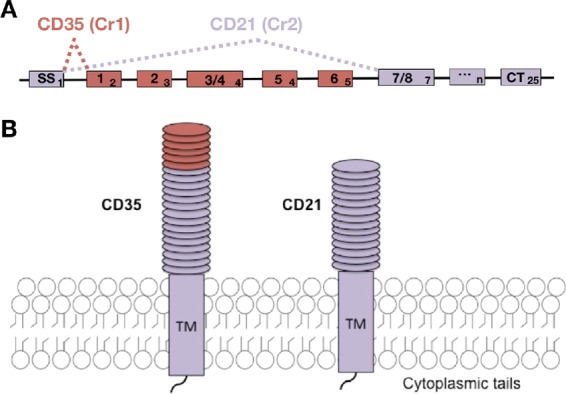
Illustration of splice variants CD35 and CD21. (A) Boxes show exons (subscript number) encoding CD21/35 elements (regular case). Murine *Cr2* encodes 25 exons, and CD21 and CD35 share exon 1 (SS_1_). Transcripts encoding CD21 splice exon 1 to exon 7, whereas CD35 includes intervening exons. (B) CD21 and CD35 both contain extracellular short consensus repeats, a transmembrane domain, and a short cytoplasmic tail. However, CD35 contains an additional 6 N-terminal SCRs. Shared sequences and structures are indicated in purple, and red indicates components unique to CD35. SS, signal sequence; CT, cytoplasmic tail.

Murine CD21/35 (CR2/1) arise from differentially splicing *Cr2* transcripts (7). Both transcripts share the first exon encoding the signal sequence, and alternative splicing to exon 7 and 2 results in CD21 and CD35 transcripts, respectively. Both CD21 and CD35 are primarily comprised of short consensus repeats (SCRs), compact repeating β-sheet domains, each approximately 60 amino acids in size and held in a coiled-coil configuration by two disulfide bonds. CD21/35 bind ligands via their SCR domains (Fig. 1A). CD21 contains 15 SCRs and recognizes C3 cleavage products, and CD35 contains an additional 6 N-terminal SCRs (Fig. 1) which also bind C4 cleavage products (7–9). Recently, transgenic mice were generated in which either CD21 (10) or CD35 (11) expression was eliminated.

Human CD21 binds to all its major ligands, iC3b, C3d, C3dg, CD23, and Epstein Barr gp350/220, using the first two SCRs, although the interaction between CD21 and CD23 additionally involves SCRs 5 to 8 (12, 13). On the other hand, human CD35 primarily binds to the complement C3b and C4b cleavage products and also to C1q (14). Some cells express both complement receptors, whereas others express only one or the other. Human CD35 expression serves to clear immune complexes by providing a phagocytic signal for macrophages (15) and neutrophils (16). CD21 forms the B cell coreceptor (BCCR) with CD19 and CD81 on B cells that provides a costimulatory signal to reduce the activation threshold upon engagement of B cell receptor with its specific antigen (17–19).

CD21/35 have been shown to facilitate prion infection administered peripherally (3–5), but the relative importance of each isoform remained unknown. Here we show that while both CD21 and CD35 bind PrP^Sc^ with high affinity, mice lacking CD21 form PrP^Sc^-replicating follicular networks closer to proximal splenic nerves, replicate PrP^Sc^ more slowly, and resist prion disease longer than mice lacking CD35. Follicular networks in CD21- and CD21/35-deficient mice appeared more fragmented and less organized and contained fewer Mfge8-positive follicular dendritic cells (FDCs) and/or tingible body macrophages (TBMφs) than wild-type (WT) and CD35-deficient mice, which could explain impaired prion pathogenesis in these mice.

## RESULTS

### CD21 and CD35 both bind PrP^Sc^.

**FIG 2:**
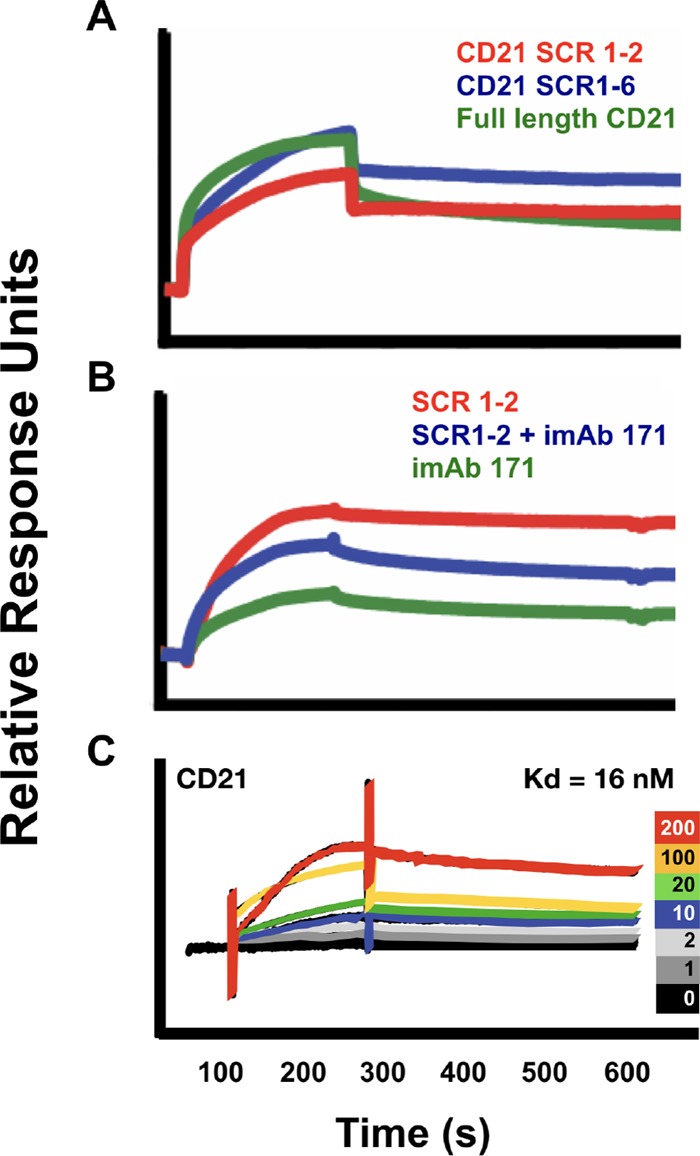
CD21 and CD35 both bind PrP^Sc^. PrP^Sc^ was enriched from elk brain infected with chronic wasting disease as previously described (21, 22). (A) Equimolar full-length CD21 or protein containing the first two or first six SCRs on CD21 bound PrP^Sc^. (B) Addition of inhibitory monoclonal antibody 171 (iMAb 171) partially reduced the interaction. (C) Kinetic analysis revealed a *K*_*d*_ of 16 nM between full-length CD21 and PrP^Sc^. Colored traces denote nanomolar ligand concentrations.

Previous reports indicate a crucial role for CD21/35 in prion disease, but whether this role can be attributed to direct interactions of CD21/35 with PrP^Sc^ remained unknown. To address this possibility, we performed a previously published protocol to remove soluble PrP^C^ and highly enrich insoluble PrP^Sc^ from infected hamster brains (20–22). We then tested whether PrP^Sc^ interacts with purified full-length CD21 or truncated CD21 containing either SCRs 1 and 2 or SCRs 1 to 6. All of these forms of CD21 bound to PrP^Sc^ with similar binding kinetics (Fig. 2A). Monoclonal antibody (MAb) 171, which binds and prevents CD21 binding to its endogenous ligands at SCRs 1 and 2 (23–25), inhibited, but did not eliminate, the interaction between CD21 and PrP^Sc^ (Fig. 2B), the dissociation constant (*K*_*d*_) of which we estimate to be 16 nM (Fig. 2C). These data suggest CD21/35 partially bind PrP^Sc^ at its first two SCRs, but may also bind PrP^Sc^ at additional SCRs. Alternatively, the prion binding site on CD21 may overlap the MAb 171 binding site, but CD21 binds PrP^Sc^ with higher affinity than MAb 171.

**FIG 3:**
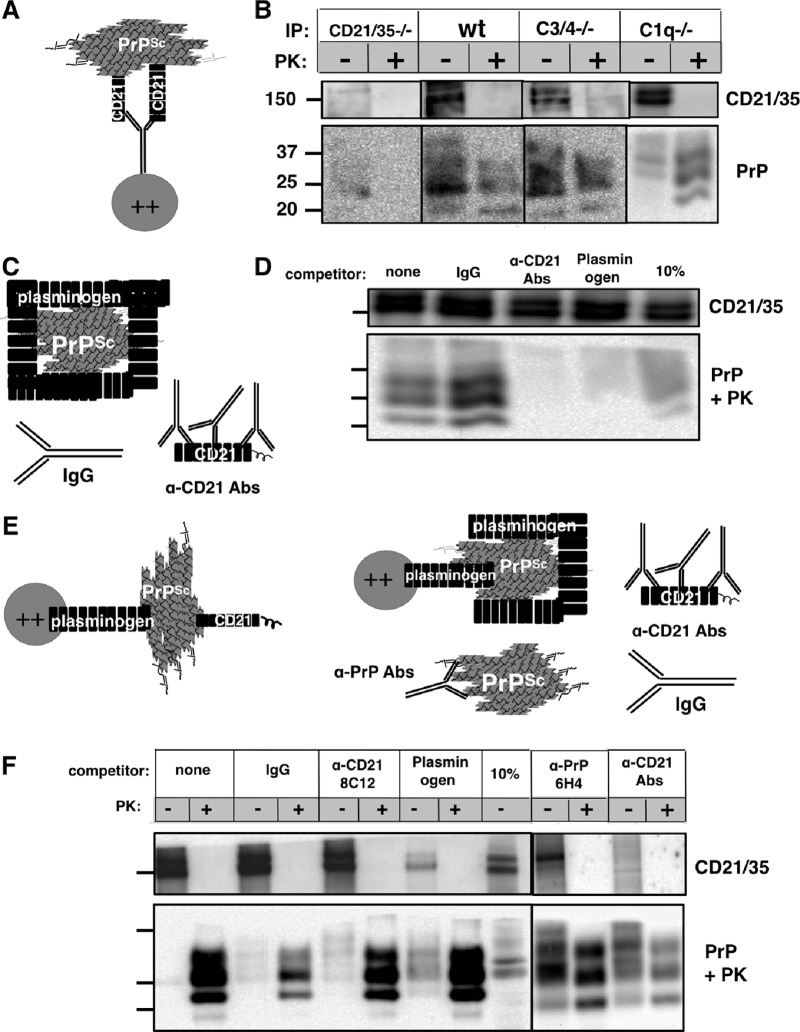
CD21/35 directly associates with PrP^Sc^. (A and B) CD21/35 coprecipitates with PrP^Sc^ with or without its endogenous complement ligands. (A) 7G6-coupled beads were incubated with C3/4^−/−^, C1q^−/−^ or CD21/35^−/−^ infected spleen homogenates, washed, and probed for PrP^Sc^. (B) Beads coated with CD21/35 precipitated PrP^Sc^ from infected spleens of all genotypes except CD21/35^−/−^ controls. Markers to the left of the blot indicate molecular mass in kilodaltons. IP, immunoprecipitation; PK, protease K. (C to F) CD21/35 can capture PrP^Sc^ *in vitro*. (C) Uninfected C3/4^−/−^ spleen-derived CD21/35-coated beads were preincubated with a cocktail of anti-CD21/35 or rat IgG2a Abs. PrP^Sc^ was preincubated with 20 µg plasminogen. (D) Excess plasminogen or the CD21/35 Ab cocktail, but not control Abs, significantly decreased capture. Ten percent input spleen homogenate and PrP^Sc^ were used as controls. Markers to the left of the blot indicate the same molecular mass in kilodaltons as in panel B. (E) Plasminogen-coated beads were incubated with 100 µg RML5, washed, and incubated with uninfected spleen homogenate from C3/4^−/−^ mice. Spleen homogenate was preincubated with 20 µg anti-CD21/35 8C12, anti-PrP 6H4 Ab, anti-CD21/35 Ab cocktail, or IgG control Ab. (F) Plasminogen-enriched PrP^Sc^ captured CD21/35, and the CD21/35 Ab cocktail and plasminogen, but not 8C12 alone or the IgG control, significantly decreased capture. The anti-PrP MAb 6H4 competed to a lesser degree. Ten percent input spleen homogenate and PrP^Sc^ were used as controls. Markers to the left of the blot indicate the same molecular mass in kilodaltons as in panel B.

To explore this further, we used paramagnetic beads coated with the anti-CD21/35 MAb 7G6, which blocks C3d binding at CD21 SCRs 1 and 2 (SCRs 7 and 8 on CD35) (26, 27), to immunoprecipitate CD21/35 from spleen homogenates from prion-infected wild-type and various complement-deficient mice (Fig. 3A) and found that PrP^Sc^ coprecipitated with CD21/35 from spleens of all mice except control CD21/35^−/−^ mice (Fig. 3B). We also used beads coated with CD21/35 from uninfected C3/4^−/−^ mice to precipitate PrP^Sc^ from prion-infected brain homogenate (Fig. 3C and D). Preincubation of CD21/35-coated beads with a cocktail of CD21 Abs, or PrP^Sc^ with plasminogen, which specifically binds PrP^Sc^ (28), abrogated CD21/35-PrP^Sc^ coimmunoprecipitation; irrelevant IgG antibodies (Abs), as control competitors, did not (Fig. 3C and D). Conversely, plasminogen-linked beads used to capture PrP^Sc^ (Fig. 3E) effectively precipitated CD21/35 from uninfected C3/4^−/−^ spleen homogenate (Fig. 3F). Excess plasminogen, but not anti-PrP or irrelevant Abs (Fig. 3E), incubated with PrP^Sc^-coated beads, successfully competed for CD21/35 binding (Fig. 3F). Anti-CD35 8C12 Ab also failed to compete for CD21/35 binding. Only a cocktail of anti-CD21/35 Abs successfully competed against PrP^Sc^ binding CD21/35, confirming that CD21/35 does not bind PrP^Sc^ exclusively to the SCRs that bind its endogenous ligands.

### sCD21 pretreatment inhibits prion infection and replication *in vitro*.

**FIG 4:**
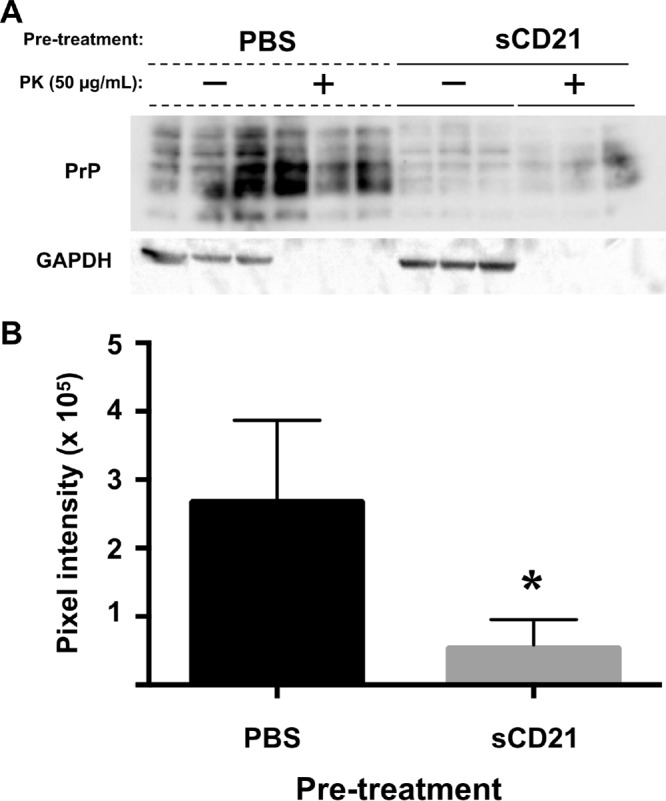
Soluble CD21 inhibits prion infection *in vitro*. RML5-infected brain homogenate (0.33%) was pretreated with either PBS or CD21 (5-µg/ml final concentration) prior to infecting N2a cells. (A) After culture of cells infected with treated prions for 4 days, cells were harvested and analyzed for PrP^Sc^ and GAPDH (glyceraldehyde-3-phosphate dehydrogenase) as a loading control. (B) Cells infected with CD21-pretreated inoculum contained significantly lower PrP^Sc^ densitometric signal than PBS-treated controls (unpaired one-way *t* test, *P* = 0.0182). Markers to the left of the blot indicate the 25-kDa molecular mass.

Identification of CD21 or -35 as a high-affinity prion receptor led us to question whether soluble CD21 (sCD21) could exert a dominant-negative effect to interfere with prion infection. Pretreatment of RML5-infected brain homogenate with CD21 led to decreased infection of and prion replication by highly susceptible N2a neuroblastoma cells compared to N2a cells infected with phosphate-buffered saline (PBS)-treated RML5 (Fig. 4).

### CD21 influences early splenic PrP^Sc^ accumulation and terminal prion disease onset.

**FIG 5:**
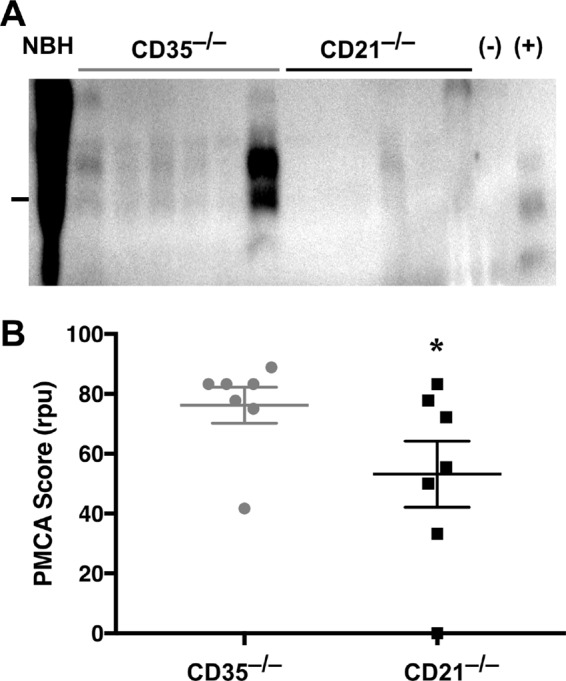
CD21 promotes splenic accumulation at 30 dpi. Mice deficient in either CD35 or CD21 were inoculated i.p. with RML5 prions and sacrificed at 30 dpi. (A) Spleen homogenates (10% [wt/vol]) were probed for PrP^Sc^ and/or subjected to serial rounds of PMCA and analyzed for PrP^Sc^ via Western blotting. The marker to the left of the blot indicates the 25-kDa molecular mass. (B) PMCA revealed that spleens from CD21-deficient mice contained significantly less PrP^Sc^ than those from CD35-deficient mice (*P* = 0.0459, one-tailed *t* test).

**FIG 6:**
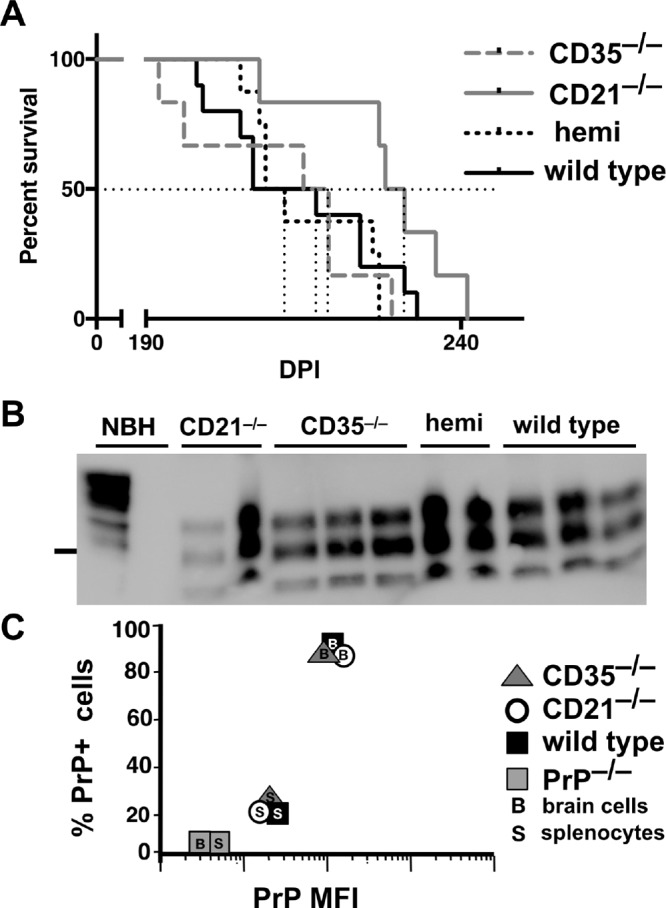
CD21-deficient mice survived terminal prion disease longer than wild-type, hemizygous, or CD35-deficient mice. Mice (*n* > 6 per genotype) were inoculated i.p. with a single high dose (10^6^ LD_50_ units) of RML5 prions and monitored for the onset of terminal disease. (A) CD21-deficient mice lived significantly longer than all other genotypes, which did not significantly differ from each other. (B) Representative Western blot confirming that all mice contained PrP^Sc^ at the onset of terminal disease. The marker to the left of the blot indicates the 25-kDa molecular mass. (C) We detected no difference in the number of PrP^+^ brain cells or splenocytes or the amount of PrP^C^ they express by FACS. Error bars do not extend beyond symbols.

We previously showed that CD21/35 expedited early splenic prion accumulation, neuroinvasion, and terminal prion disease (3, 5). To determine if one isoform preferentially facilitates this early pathogenesis, we inoculated mice with RML5 prions and assessed PrP^Sc^ loads in spleen at 30 days postinfection (dpi). Mice deficient in CD21 accumulated less PrP^Sc^ in their spleen than CD35-deficient mice (Fig. 5; *P* = 0.0459). We also monitored time to terminal prion disease in these mice to ascertain whether splice variant CD35 or CD21 promotes disease onset. We found that CD21-deficient mice resisted disease significantly longer than wild-type, CD35-deficient, or hemizygous mice (Fig. 6; Table 1), although the levels of PrP^Sc^ accumulation in their brains at terminal disease appeared similar (Fig. 6B). We detected no difference by fluorescence-activated cell sorting (FACS) in the number of PrP^C^-positive brain cells or splenocytes or the amount of PrP^C^ those cells express to account for the differences in prion disease kinetics we observed (Fig. 6C).

**TABLE 1:** Terminal prion disease progression

Genotype^a^	Incidence^b^	Mean dpi ± SD^c^
WT (C57BL/6)	17/17	212 ± 12^d^
Hemizygous	8/8	215 ± 10
CD35^−/−^	6/6	212 ± 14
CD21^−/−^	6/6	229 ± 11^e^
CD21/35^−/−^	10/10	255 ± 26^d,e^

a Mice were inoculated i.p. with 10^6^ LD_50_ units of RML5 prions.

b Number of terminally ill mice/number of mice inoculated.

c Days postinoculation ± standard deviation.

d Data from our previous study (2) are included in these analyses.

e *P* < 0.05 compared to all other groups.

### CD21 alters CD19 expression and follicular networks.

**FIG 7:**
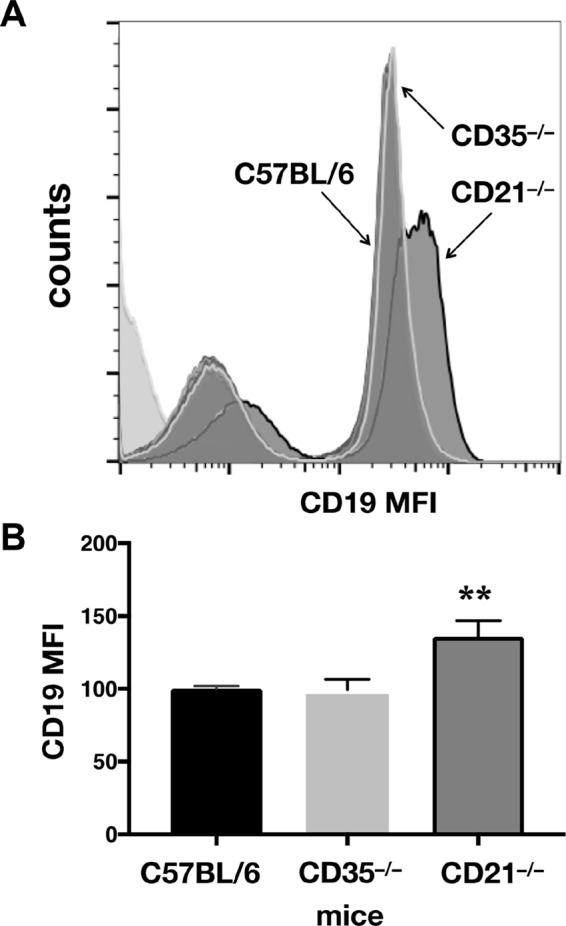
CD21 deficiency increases CD19 expression. (A) Flow cytometry revealed an increase in CD19 expression on B cells from CD21-deficient mice compared to CD35-deficient and wild-type mice. (B) CD19 MFI was statistically significantly higher on CD21-deficient B cells (**, *P* < 0.01).

**FIG 8:**
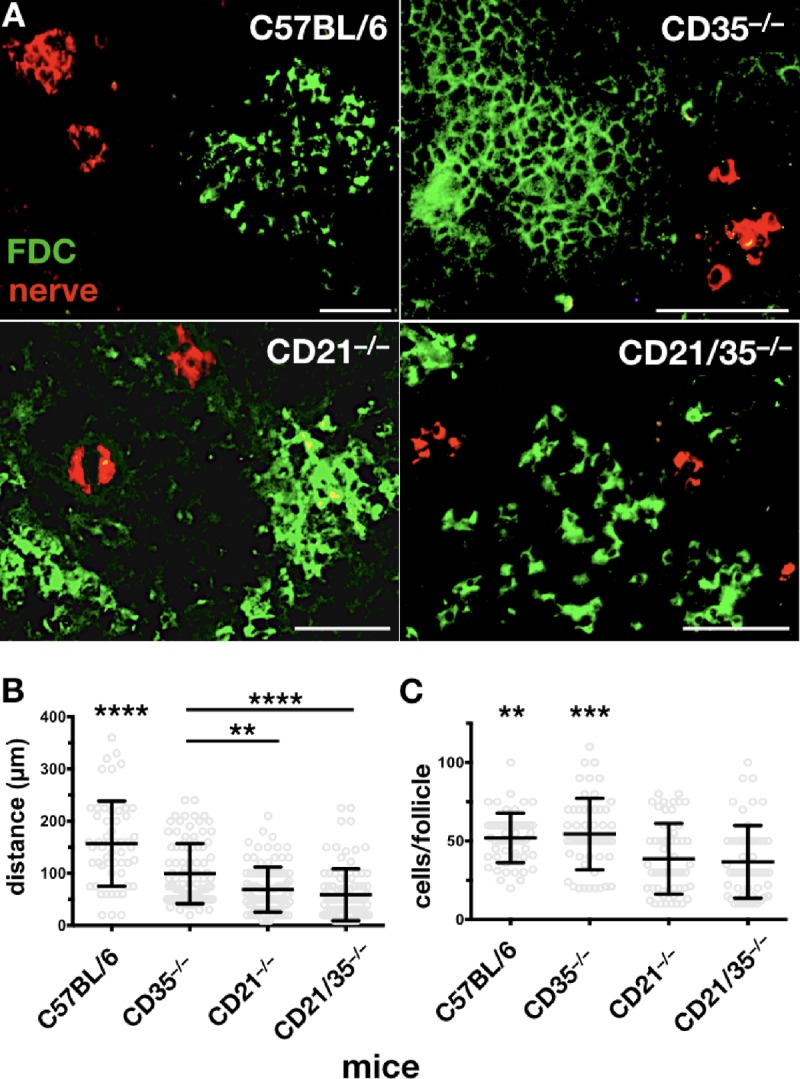
CD21/35 affects follicular development. (A) Immunofluorescent histology of 5-µm frozen spleen sections stained with anti-Mfge8 Ab to identify follicles (FDCs and TBMφs [green]) and anti-tyrosine hydroxylase (anti-TH) Ab to identify nerve fibers (red). Proximal nerves appeared closer to follicular networks in spleens from CD21-, CD35-, and CD21/35-deficient mice than wild-type C57BL/6 mice. Follicular networks appeared more fragmented and less organized, with fewer Mfge8-positive FDCs and TBMφs forming networks in CD21- and CD21/35-deficient spleens than in CD35-deficient or wild-type spleens. (B) Mean neurofollicular distances were shortened in the absence of CD21 (*n =* 95), CD35 (*n =* 92), or CD21/35 (*n =* 94), with no statistically significant difference between distances observed in CD21- and CD21/35-deficient spleens. Distances measured in C57BL/6 spleens (*n =* 57) were significantly different from those of all other groups. ****, *P* < 0.0001; **, *P* < 0.01. (C) CD21 (*n =* 54)- and CD21/35 (*n =* 52)-deficient splenic follicles contained significantly fewer Mfge8-positive FDCs and TBMφs than CD35-deficient (*n =* 50) and C57BL/6 (*n =* 54) follicles. **, *P* < 0.01, and ***, *P* < 0.0001, compared to CD21- and CD21/35-deficient follicles.

**FIG 9:**
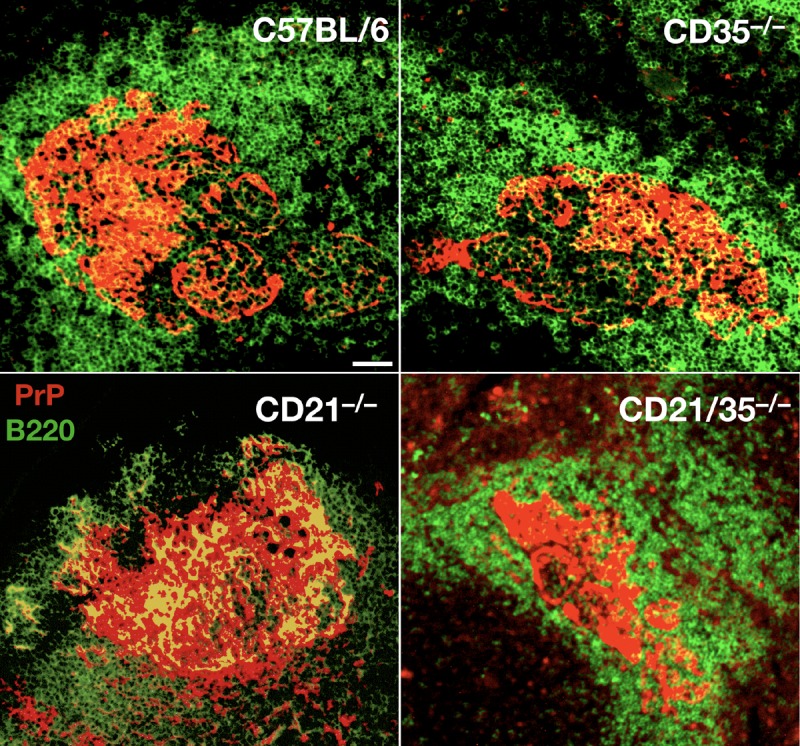
Normal B cell architecture and PrP^C^ expression in mice deficient in CD21, CD35, or both. Immunofluorescent staining of splenic follicles for PrP^C^ (red) and B220 (green) showing relatively normal B cell numbers and structure and comparable PrP^C^ expression in follicles from wild-type (C57BL/6), CD35^−/−^, CD21^−/−^, and CD21/35^−/−^ spleens. We observed slightly expanded marginal zone B cells in CD21/35^−/−^ spleens, but total B cell numbers were not significantly altered. Scale bar, 100 µm.

Previous reports indicate that lack of CD21/35 leads to increased expression of another member of the BCCR, CD19 (29, 30). B cells from CD21/35-deficient mice express 33 to 49% more CD19 than those from CD21/35-sufficient mice. Additionally, changes in CD19 expression have been shown to impact prion disease. CD19-deficient mice exhibit accelerated prion neuroinvasion, most likely due to their forming FDC networks that replicate PrP^Sc^ in spleens closer to adjacent nerve fibers (31). To determine whether CD21/35 could be indirectly affecting prion pathogenesis by altering CD19 expression and/or function, we first investigated CD19 expression on CD21- and CD35-deficient mice. Flow cytometry revealed a significant 38% increase of CD19 on B cells from CD21-deficient mice compared to wild-type or CD35-deficient mice (Fig. 7). Since CD19’s absence results in FDC networks closer to proximal nerves, we investigated whether CD19 upregulation positions FDC networks farther from them. We found that, like CD19-deficient mice, CD21- and CD21/35-deficient mice also contain splenic follicles positioned closer to proximal nerves than wild-type or CD35-deficient mice (Fig. 8A and B). Thus, despite reduced neurofollicular distances in their spleens, CD21-deficient mice still survived prion infection longer than wild-type or CD35-deficient mice, coinciding with the reduced splenic PrP^Sc^ replication we observed early after infection. Efficient prion replication in lymphoid follicles requires intact, organized follicular networks where PrP^C^ and CD21/35-expressing FDCs and B cells can efficiently capture and replicate PrP^Sc^. We observed more fragmented, less organized follicular networks composed of significantly less FDCs and TBMs in spleens from CD21- and CD21/35-deficient mice than CD35-deficient and wild-type mice (Fig. 8A and C), which could explain their differential prion replication. We observed little or no differences in B cell architecture and PrP^C^ expression, save slightly increased marginal zone B cells in CD21/35^−/−^ spleens (Fig. 9) as previously described (30).

## DISCUSSION

Attempts to ascertain CD21/35 functions previously relied on *Cr2* genetic manipulations that render mice deficient in both CD21 and CD35. However, Donius et al. (10, 11) generated mice that express one splice variant or the other, allowing researchers to assess the function and relative importance of each splice variant to cells that express them. For example, CD35 on macrophages and neutrophils is generally thought to promote phagocytosis and immune complex clearance, whereas CD21 on follicular dendritic (FDC) and B cells is thought to enhance antigen presentation and adaptive immune responses, respectively. These mice offer the ability to challenge or confirm these proposed roles.

The role of complement receptors in prion disease is well established. Mice deficient in CD21/35 exhibit delayed clinical disease when challenged with mouse-adapted scrapie prions (3) or an elk-derived isolate of chronic wasting disease prions (5). These seminal papers highlight a crucial role in CD21/35 in prion disease, although the relative importance of each splice variant remained unknown.

Here we show that both variants derived from *Cr2* transcripts can biochemically interact with PrP^Sc^ (Fig. 2). This finding supports the previous *in vivo* data and suggests CD21/35 are cell-surface prion receptors. Interestingly, in the mouse model of chronic wasting disease, C3-deficient mice eventually succumbed to terminal prion disease, whereas CD21/35-deficient mice did not (5, 6). Our current data support the idea that CD21/35 impacts prion disease by directly interacting with PrP^Sc^ independent of its endogenous complement ligands. These data suggest that CD21/35 binds PrP^Sc^ in a less-site-specific manner than its endogenous ligands, but do not exclude the possibility of CD21/35 binding to C3 and C4 cleavage products bound PrP^Sc^. CD21/35 likely exerts its effects along with C3/4 opsonization, although perhaps not crucially, nor do our current data exclude the remote possibility that other proteins mediate this interaction.

We also confirmed CD21/35 as prion receptors and that soluble CD21 (sCD21) can act in a dominant-negative fashion to inhibit prion replication and infection of N2a cells in cell culture. Cells infected with prions pretreated with CD21 appeared to contain less total PrP signal (without protease K [PK] digestion) than PBS-treated controls. Prion replication likely increased the PrP^Sc^ signal that contributed to the higher total PrP signal in PBS-treated samples, but we cannot definitively discount the possibility that CD21 caused a reduction of PrP^C^. In either case, these data suggest sCD21 could serve as a therapeutic, and we are currently exploring the use of sCD21 or soluble SCRs as small, prion-inhibitory molecules *in vivo*.

CD21/35-expressing B cells and FDCs are known to impact prion disease. B cells likely promote prion trafficking more so than replication, because deletion of *prnp* specifically in B cells did not inhibit prion disease (32, 33). Thus, B cells promote prion disease independent of PrP^C^ expression, and the data presented here support that hypothesis. We have previously shown that optimal prion trafficking to, capture by, and replication in lymphoid follicles require CD21/35 expression on FDCs and B cells (3, 5). We propose CD21 acts as the prion receptor on B cells that mediates these facets of prion pathogenesis.

FDCs previously have been shown to be perhaps the most important immune cell type for peripheral prion pathogenesis. They express the most PrP^C^ of any immune cell, and ablating them or manipulating follicular networks impacts peripherally initiated prion disease more than any other cell type (34, 35). However, B cells help orchestrate follicular development by providing lymphotoxin α and β as maturation and maintenance signals to FDCs (35, 36). Loss of the BCCR member CD19 alters follicular development, moving FDC networks closer to proximal nerves and expediting neuroinvasion and terminal prion disease (31). We observed the same phenomenon in CD21- and CD21/35-deficient and, to a lesser extent, CD35-deficient spleens. However, despite reduced neurofollicular distances in CD21-deficient mice, they still survived prion disease significantly longer than wild-type and CD35-deficient mice. CD21-deficient mice express full-length CD35 and a slightly truncated form missing SCRs 5 and 6 (11), which may not bind prions as efficiently. However, CD35-deficient mice also lack these and another four SCRs, and our SPR and coimmunoprecipitation data showing prions binding multiple SCRs argue against this explanation. CD21- and CD35-deficient mice develop normal splenic architecture, express normal populations and proportions of B cells, and express normal levels of PrP^C^. CD21/35 mice do express slightly expanded marginal zone B cells (30), but one would expect that expansion to result in enhanced prion replication, if any effect at all. We did observe fragmented, less organized follicles that contained less Mfge8-positive FDCs and TBMφs in spleens from CD21- and CD21/35-deficient mice that we conclude impaired early prion capture, replication, and eventual neuroinvasion. CD21 forms the BCCR with CD19 and CD81 and appears to be the more important isoform for proper follicular development. Along with altered FDC networks observed in CD19-deficient mice, these data strongly promote the BCCR as an important signaling complex in developing normal follicular architecture. We conclude that proper follicular development and organization require CD21, leading to more efficient lymphoid prion replication and expedited neuroinvasion in CD21-expressing mice than in mice expressing the CD35 isoform.

Known CD35 functions include phagocytosis and clearance of opsonized pathogens and immune complexes on neutrophils and macrophages (15, 16). CD35 can also biochemically interact with PrP^Sc^, but likely promotes its phagocytosis by and destruction and/or sequestration within macrophages, the only cell type known to help resolve prion infection (37). CD21/35 likely act as PrP^Sc^ receptors on FDCs, which act as efficient PrP^Sc^ bioreactors in the lymphoreticular system. CD21, however, engages C3d/g opsonized pathogens and provides a costimulatory signal when a mature, naive B cell encounters its specific antigen. Therefore, in conjunction with previous reports, our data clarify the role of B cells in prion disease, including promotion of optimal follicular development mediated by the BCCR and interaction of CD21/35 with PrP^Sc^.

## MATERIALS AND METHODS

### Mice.

All mice were bred and maintained at Lab Animal Resources, accredited by the Association for Assessment and Accreditation of Lab Animal Care International and approved on 14 January 2016 by the Institutional Animal Care and Use Committee at Colorado State University (protocol ID 09-1580A). CD21 (CR2)- or CD35 (CR1)-specific knockout mice on the C57BL/6 background were generated and characterized as previously described (10, 11). We crossed the individual knockout mice to achieve hemizygosity.

### Mouse inoculations.

Age- and sex-matched mice (*n* ≥ 6 per genotype) ranging in age from 6 weeks to 1 year intraperitoneally (i.p.) received 100 µl of approximately 10^6^ 50% lethal dose (LD_50_) units of mouse-adapted scrapie strain RML5 prions (38).

### Clinical sign scoring.

Mice were monitored daily and sacrificed at the onset of terminal disease or specified time points. We employed a scoring system to assess the severity of disease, including: tail rigidity (0 to 2), akinesia (0 to 4), ataxia (0 to 4), tremors (0 to 4), and weight loss (0 to 2). Mice that scored above 10 were euthanized via CO_2_ inhalation, replacing 20% of air per minute to effect.

### Tissue collection and analysis.

After euthanasia, the following samples were collected and frozen or fixed in 4% formaldehyde in PBS: serum, spleen (half fixed, half frozen), kidneys (one fixed, one frozen), tail clip, and brain (half fixed, half frozen). We assessed the presence of PrP^Sc^ in 10% (wt/vol) homogenate after protease K (Roche) digestion (10 µg/ml for spleen and 50 µg/ml for brain) and Western blotting using anti-PrP monoclonal antibody (Ab) BAR 224 (Cayman Chemical) conjugated to horseradish peroxidase (HRP). Blots were developed using chemiluminescent substrates hydrogen peroxide and luminol for 5 min at room temperature and visualized using a GE digital imager and ImageQuant software. Tissues negative for PrP^Sc^ on Western blots were subjected to serial protein misfolding cyclic amplification (PMCA [39]). Briefly, we used 10% normal brain homogenate (NBH) in PMCA buffer (PBS, 1% Triton X-100, 4 mM EDTA, 150 mM NaCl) from PrP^C^-overexpressing transgenic mice of strain Tga20 (40) as the substrate for amplification of previously undetectable PrP^Sc^. Twenty-five microliters of NBH and 25 µl 10% sample homogenate were sonicated for 40 s at ~150 W, followed by a 30-min incubation, which was repeated for 24 h (one round). Serial rounds were performed similarly, transferring 25 µl of the previous round’s sample to 25 µl of fresh NBH. Each biological sample was run in at least technical duplicates, and round to positivity was determined by protease K (PK) digestion and Western blotting. Relative PMCA units were assigned as previously described (41).

### PrP^Sc^ enrichment.

PrP^Sc^ was enriched from infected brain as previously described (20, 21). Briefly, a brain from an elk infected with chronic wasting disease (E2) was homogenized in PBS to a 10% (wt/vol) concentration. Sucrose (1.2 M) was added to 10 ml of clarified tissue to a final concentration of 165.5 mM, and samples were ultracentrifuged (100,000 × *g*) for 1 h at 4°C. Pellets were resuspended to a final protein concentration of 5.0 mg/ml in 1× Tris-buffered saline (TBS) containing 2.0% Triton X-100 (TBST) and incubated on ice for 30 min. Samples were subjected to another round of ultracentrifugation for 20 min at 0°C and washed twice in 1× TBST and twice with 1× TBS. The pellets were then resuspended in PBS containing 1% Sarkosyl and protease inhibitor cocktail (Roche). Vortexed samples were incubated on a heated shaker at 37°C for 2 h at 800 rpm. Samples were then gently overlaid onto a cushion of 0.32 M sucrose in PMCA buffer 1 (PMCA buffer without Triton X-100) and ultracentrifuged for 1 h at 4°C, and supernatants were removed. Pellets were resuspended in 2.3 M NaCl–5% Sarkosyl in PBS, centrifuged at 13,000 × *g*, washed three times in 50 mM Tris–150 mM NaCl, and either stored dry or suspended in PBS at −80°C. The presence of PrP^Sc^ was confirmed by Western blotting. Lack of complement proteins in these preparations was confirmed by enzyme-linked immunosorbent assay (ELISA).

### Expression of recombinant wild-type CD21 SCR1 to -6 and CD21 SCR1 and -2.

The codon-optimized (Homo sapiens) DNA sequences for human CD21 SCR1 to -6 and CD21 SCR1 and -2 (comprising residues 21 to 409 and residues 21 to 153, respectively; Uniprot identifier P20023), which had been subcloned into the pDONR221 entry vector (Life Technologies, Inc.) were purchased from GeneArt. The engineered sequences additionally contained DNA encoding an Ig kappa chain leader sequence to facilitate secretion of the target protein, a Gly-Ala-Gly-Ala-Gly-Ala linker region, a hexahistidine (His_6_) fusion tag, a second linker region (Asp-Tyr-Asp-Ile-Pro-Thr-Thr), and a tobacco etch virus (TEV) nuclear inclusion A endopeptidase cleavage site (Glu-Asn-Leu-Tyr-Gln-Gly), all of which are located 5′ prime to the synthetic genes. These sequences were then recombined into a pcDNA3.2/V5-Dest expression vector using Gateway LR clonase II enzyme mix (Life Technologies, Inc.) according to the manufacturer’s instructions. The resulting expression constructs were amplified and transiently transfected into Freestyle 293f cells grown in suspension in an 8% CO_2_ humidified environment at 37°C. After 96 h, the cells were harvested by centrifugation, and the spent medium was collected and passed through a 0.22-μm-pore filter to remove cellular debris.

CD21 SCR1 to -6 were prepared by diluting spent medium into a 5× buffer containing 0.1 M sodium phosphate (pH 7.8), 2.5 M NaCl, 0.1 M imidazole (to give a final working concentration of 20 mM sodium phosphate [pH 7.8], 0.5 M NaCl, 20 mM imidazole). The diluted protein was then applied to a 5 ml Histrap HP column (GE Healthcare, Inc.) using an ÄKTAprime Plus (GE Healthcare, Inc.) liquid chromatography system and subsequently eluted using a linear imidazole gradient (20 mM to 0.5 M). CD21 SCR1 to -6 were then concentrated at room temperature using a Vivaspin 20 device (Millipore Inc.) and applied to a HiPrep 16/60 Sephacryl S200 size exclusion column (GE Healthcare, Inc.) that had been equilibrated with Dulbecco’s phosphate-buffered saline (DPBS) without MgCl_2_ or CaCl_2_ (DPBS is 2.7 mM KCl, 1.5 mM KH_2_PO_4_, 138 mM NaCl, 8.1 mM Na_2_HPO_4_ [pH 7.4]) using an ÄKTADesign high-pressure liquid chromatography system (GE Healthcare, Inc.). The purity of the resulting purified recombinant CD21 SCR1 to -6 was assessed by SDS-PAGE.

CD21 SCR1 and -2 were also initially purified from spent medium by immobilized metal affinity chromatography in a manner identical to that described for CD21 SCR1 to -6. However, after elution, the partially purified CD21 SCR1 and -2 were buffer exchanged into a 1/3 DPBS buffer and applied to a C3d affinity column, generated by binding glutathione *S*-transferase (GST)-tagged C3d to a 5-ml GSTrap column (GE Biosciences). CD21 SCR1 and -2 were eluted using a linear NaCl gradient of 0 to 0.5 M in 1/3 PBS as previously described (42). Any coeluted GST-C3d was removed by manually applying the resulting solution to a 1-ml GSTrap column. Finally, purified CD21 SCR1 and -2 were concentrated in a Vivaspin 20 concentration device and assessed by SDS PAGE.

### Production of the anti-CR2 iMAb 171.

The anti-CR2 inhibitory monoclonal antibody (iMAb) 171 was obtained from the spent culture medium of hybridoma cells grown in RPMI 1640 supplemented with 2 mM l-glutamine, 100 IU penicillin, 100 µg/ml streptomycin, and 10% fetal bovine serum. Abs were purified by affinity chromatography using protein G Sepharose 4 Fast Flow resin (GE Healthcare Biosciences Corp.). Purified iMAb 171 was then exchanged into PBS and finally concentrated to give a stock solution containing 1 mg/ml.

### SPR.

Highly enriched PrP^Sc^ were coupled to CM5 sensor chips outside the instrument after generating the reference flow cells within the instrument by activation with EDC/NHS [0.2 M 1-ethyl-3-(3-dimethylaminopropyl) carbodiimide hydrochloride and 0.05 M *N*-hydroxysuccinimide] and deactivation with ethanolamine three to five times. The chip was then removed from the instrument and the gold chip disassembled from the cassette. The entire surface was activated with 100 µl of EDC/NHS for 12 min. A PrP^Sc^ pellet was resuspended in 100 µl of 10 mM sodium acetate (pH 5.4), sonicated at 37°C for 40 s, and incubated on the gold chip at room temperature for 1 h. The chip was then briefly rinsed with PBS, and the remaining active groups were deactivated with ethanolamine for 7 min. Prior to use in interaction analyses, a startup cycle of 50 mM sodium hydroxide served to remove any nonspecifically bound PrP^Sc^ from the surface.

All surface plasmon resonance (SPR) experiments involved recombinant human PrP (rPrP) or PrP^Sc^ enriched from infected elk brain as the ligand coated to a CM5 series S sensor chip. Purified CD21 and CD21 containing SCR1 and -2 or SCR1 to -6 was buffer exchanged in Amicon filter devices into 1× running buffer (50 mM Tris HCl, 150 mM NaCl [pH 7.42]). The rPrP-coated chip was kindly provided by Hae-Eun Kang in the Telling lab. Briefly, flow cells were first activated with EDC/NHS for 7 min at 10 µl/min. Amine coupling of recombinant cervid or murine PrP^C^ in 10 mM sodium acetate (pH 5.5) was accomplished by flowing 20 µg/ml of ligand over the activated chip for 7 min at 10 µl/min. Excess activated groups were deactivated with 1 M ethanolamine HCl (pH 8.5) for 7 min at 10 µl/min. Reference flow cells, built-in negative controls for this system, underwent rounds of activation and deactivation without protein ligand.

### Cell culture and *in vitro* prion infection.

N2a mouse neuroblastoma cells were grown in RPMI 1640 medium containing 10% fetal bovine serum and 1% penicillin–streptomycin. RML5-infected brain homogenate was UV sterilized prior to infection of cells. RML5 was preincubated with PBS or CD21 (SCR1 and -2; 5 µg/ml final concentration) for 10 to 20 min prior to infecting cells. N2a cells were seeded at 100,000 cells per well in a 12-well plate and infected with 0.3% RML5. Cells were grown at 37°C and 5% CO_2_ for 4 days. Wells were rinsed 2× in PBS, and cells were detached from the plate using 5 mM EDTA in PBS for 10 min. Cell pellets were resuspended in 100 µl PMCA buffer containing 1% Triton X-100 and lysed on ice for 30 min. Lysates were assessed for PrP^Sc^ using traditional PK digestion and Western blotting techniques.

### FACS.

Fresh brain and spleens were harvested and processed to single-cell suspensions in 3 ml PBS. Briefly, tissues were passed through 40-µm-pore mesh filters using sterile plungers and cold PBS washes. Cells were pelleted at 250 × *g* for 5 min at 4°C, and the supernatant was discarded. Red blood cells (RBCs) were lysed in ACK (ammonium-chloride-potassium) buffer for 5 min, and the remaining cells were pelleted and washed with FACS buffer (PBS, 1% fetal bovine serum [FBS], 150 mM EDTA). Primary splenocytes or brain suspensions were blocked in 7% mouse serum and 1:50 Fc block (BD Pharmingen) for 20 to 60 min on ice. Ab solutions (1:100 final) were added to cells and incubated in the dark for 1 h on ice. Cells were washed by adding 1 ml FACS buffer to existing Ab solution. Cells were pelleted and resuspended in 1 ml FACS buffer for a total of 3 washes. A 792-µl cell suspension was added to 8 µl of 100 µg/ml propidium iodide (Sigma) immediately prior to data acquisition in a Cyan flow cytometer. Unstained samples were analyzed first to set detector voltage and gating parameters to place the mean fluorescent intensity of at least 99% of unstained cells in a well-defined peak in the first decade of a log scale. Mean fluorescent intensity (MFI) signals beyond this decade were called positive. MFI as well as frequency of parent gate values were imported into Excel and/or GraphPad Prism for analysis.

### PAA and immunoprecipitations.

The PrP^Sc^ affinity assay (PAA) was performed as previously described (28). Sixty micrograms of CD21/35 Ab 7G6 or 7E9 (BD Pharmingen) was incubated with 10^9^ M-270 magnetic epoxy beads (Dynal, Oslo, Norway) in 1 M ammonium sulfate in PBS for 72 h at 4°C with tilt rotation. Beads were washed with PBS and then blocked overnight at 4°C with 5% bovine serum albumin (BSA) in PBS, washed, and resuspended in 500 µl PBS. Anti-CD21/35 or plasminogen beads were incubated with 1,000 µg infected or uninfected spleen homogenate overnight at 4°C or 100 µg RML5 for 90 min at room temperature, respectively. CD21/35- or PrP^Sc^-coated beads were washed and incubated with 100 µg RML or 1,000 µg uninfected spleen homogenate, respectively. For competition experiments, free or plasminogen bead-bound RML5 was incubated overnight at 4°C with 20 µg plasminogen, mouse IgG, or anti-PrP MAb 6H4. Spleen homogenate or immunoprecipitated CD21/35 was incubated overnight at 4°C with 20 µg anti-CD21/35 Ab 7G6 (Pharmingen) or a cocktail of anti-CD21/35 Abs consisting of 7G6, 8C12, 7E9, 8D9 (Pharmingen), and D-19 (Santa Cruz).

### Immunofluorescent histology and morphometry of spleen sections.

Spleens were removed from mice of each genotype and flash frozen in OCT medium in liquid nitrogen. Five-micrometer sections were cut onto glass slides, fixed in ice-cold acetone for 10 min, air dried overnight, and incubated in 1:50 dilution of Fc block (BD Pharmingen) and 10% rat serum in Ultra Cruz blocking reagent (Santa Cruz) for 1 h at room temperature. Samples were then incubated in anti-mouse tyrosine hydroxylase (TH) Ab, rinsed three times for 5 min with PBS, followed by incubation with CruzFluor 555 (CF555)-conjugated mouse IgG binding protein (Santa Cruz) to stain splenic nerves. Slides were then rinsed and blocked again and then incubated with anti-mouse Mfge8 Ab followed by CF488-cojugated mouse IgG binding protein (Santa Cruz) to stain follicular dendritic cells (FDCs) and tingible body macrophages (TBMφs). B cells were stained with Alexa 488-conjugated anti-B220 Ab (Pharmingen). PrP^C^ was detected using Alexa 650-conjugated BAR 224. Slides were rinsed again and coverslips mounted with ProLong Gold antifade mounting medium (Life Technologies, Inc.). Splenic follicles were visualized with an Olympus BX-60 fluorescence microscope, and images were captured using a DP-71 charge-coupled diode camera (Olympus). Neurofollicular distances were measured and cells counted in at least six nonconsecutive sections from two spleens from two mice of each genotype using a morphometric overlay module in GraphicConverter (Lemke Software).

### Statistical analyses.

All statistical analyses were performed using GraphPad Prism software. We used log rank tests to compare survival curves, Student’s *t* test to compare PMCA scores, and one-way analyses of variance (ANOVA) for all other comparisons among genotypes. Data comparisons with *P* values of <0.05 were considered significantly different. Technical duplicates were averaged, and the mean value of each biological replicate was considered an *n* value of 1.
